# Post-operative pain and success rate after pulpotomy with symptomatic irreversible pulpitis: A review

**DOI:** 10.6026/973206300210196

**Published:** 2025-02-28

**Authors:** Omar Al Jasir

**Affiliations:** 1 Department of Conservative Dental Science, College of Dentistry, Qassim University, Kingdom of Saudi Arabia

**Keywords:** Post-operative pain, pulpotomy, cariously exposed, permanent molars, irreversible pulpitis

## Abstract

It has long been assumed that permanent teeth with a diagnosis of "irreversible pulpitis" had an irreparably damaged dental pulp. Pulpotomy, a crucial pulp treatment operation that is frequently disregarded, has recently resurfaced as a less invasive procedure. In endodontics, promoting less invasive procedures aimed at preserving pulp vitality has emerged as a top objective. Permanent teeth with exposed carious pulp can be effectively treated with pulpotomy. Therefore, it is of interest to review the success rate and post-operative pain following pulpotomy in adult permanent molars that are cariously exposed.

## Background:

In addition to producing pathological defense responses to traumas, caries and surgical treatments, the dentin/pulp complex experiences physiological changes throughout its life under normal conditions [1]. The basic goal of a partial or complete pulpotomy procedure is to permanently remove inflammatory pulp tissue while leaving the remaining normal or reversibly inflamed pulpal tissues intact [2]. Partial or complete pulpotomies, as opposed to endodontic therapy, maximize the preservation of living pulp tissue to maintain its potent sensory, defensive, nutritional and regenerative properties, hence adhering to the idea of minimal invasion [1]. A vital pulp treatment (VPT) operation called pulpotomy is frequently contemplated for mature permanent teeth that show signs of irreversible pulpitis. In teeth with carious pulp exposure, it is being used more and more as an alternative to root canal therapy (RCT) [3]. In their randomized controlled investigation, Zhu *et al.* came to the conclusion that full pulpotomy (FP) would be a suitable substitute treatment for managing mature teeth with irreversible pulpitis (IP) [3]. In the past, pulpotomy was the gold standard for treating mature permanent teeth with symptomatic or asymptomatic irreversible pulpitis, whereas pulpotomy was chosen for deciduous and immature permanent teeth [4]. A full, coronal, or complete pulpotomy entails "complete removal of the coronal pulp and the application of a biomaterial onto the pulp tissue at the level of the root canal orifice(s)" [5] according to the European Society of Endodontology (ESE, 2019) [6].

According to Fuks (2008), the residual pulp can heal with the right wound dressing and tooth restoration if tissue removal during partial or complete pulpotomy is prolonged to a point when the underlying tissue is either not inflamed or reversibly inflamed [5, 7]. Since younger pulp is more vascular, cellular and has greater reparative potential, pulpotomy is typically advised for younger individuals [8]. Ricucci *et al.* 2014 and 2019 showed histological evidence that reversible or irreversible pulpitis is a part of a pulpal condition and not representative of the entire pulp tissue. By removing the diseased pulpal tissue, vitality of the pulp can be maintained with VPT [9, 10]. Furthermore, the tooth becomes susceptible to further lesions when the pulp's defensive, dentinogenic and sensory capabilities are lost [11]. When compared to pulpectomy and root canal therapy, pulpotomies are thought to be more economical, less time-consuming and technically simpler [12]. Therefore, it is of interest to review the success rate and post-operative pain following pulpotomy in adult permanent molars that are cariously exposed.

## Efficacy of complete pulpotomy:

Recent studies have shown promising success rates for pulpotomy procedures in mature permanent teeth. For instance, Ramani *et al.* [13] reported an 89.8% success at one year. Similarly, another recent study by Jassal *et al.* has also suggested that at 1-year follow-up, the success rate was 91.6% [14]. Furthermore, a randomized clinical trial by Taha *et al.* indicated a 93% success rate and concluded that; pulpotomy has favorable outcomes for mature teeth [15]. It was noted that the pulpotomy group's patients expressed much greater satisfaction and thought the procedure was enjoyable. This could be caused by the shorter procedure of pulpotomy; whereas RCT is a longer procedure. This finding is corroborated by their study in (2022) when performed complete pulpotomy procedure using 3 calcium silicate-based materials, which noted that overall success at 1 year was 92.3% [15]. Another randomized clinical study by Galani *et al.* (2017) also supports these findings; demonstrating that VPT, including pulpotomy, yields overall success rates of 85% clinically as well as radiographically at the end of 18 months follows up [16]. However, this study justified the failure of pulpotomy in 3 cases was due to limitations of available diagnostic aids to correctly diagnose pulpal disease. Although the results are based on diverse studies with a significant risk of bias, a recent systematic analysis indicates that pulpotomy has a high success rate for teeth exhibiting permanent pulpitis signs and symptoms [16]. More research involving a control group of teeth that received root canal therapy and longer follow-up times are required, but overall, full coronal pulpotomy had a good success rate in treating carious critical pulp exposure of permanent adult teeth with closed root apices [17].

## Post-operative pain:

The primary subjective symptom that drives a patient to seek endodontic therapy is pain. The primary predictor of postoperative pain is the existence of preoperative discomfort [18]. The success of a specific treatment strategy in managing pain is reflected in postoperative pain episodes and the need for analgesics [19]. The Visual Analogue Scale was used to compare how much pain decreased over time. According to Galani *et al.* the pulpotomy group experienced greater symptom reduction, with 70.3% of patients reporting pain on the first day [16]. But there was little pain, thus no analgesic was required. Pulpotomy can therefore be considered an alternate treatment for pain alleviation in an emergency. These results are consistent with those of Ramani *et al.* [13]. The complete pulpotomy (CP) group experienced considerably lower pain intensities and analgesic consumption, suggesting that CP was a more effective approach of achieving the short-term goal of pain control [20]. In both groups, there was a noteworthy decrease in postoperative pain scores at 24, 48 hours and 7 days. A decrease in the pulp chamber's local pressure and degree of inflammation may be the cause of the pain relief during pulpotomy. According to the findings of these earlier investigations, pulpotomy can relieve pain just as well as pulpectomy [13].

## Level of pulpal biomarkers:

Clinicians currently use a variety of subjective methods, such as history, examination and pulp sensibility, to determine the inflammatory condition of pulp tissue. Previous research has shown that pulpitis is associated with an increase of a number of markers, including matrix metalloproteinases (MMPs) and inflammatory cytokines [21]. For carious teeth with irreversible pulpitis, pulpotomy is regarded as the final treatment option. The possibility of pulpotomy in treating teeth with pulpitis is gaining momentum with the growing popularity of minimally invasive dentistry world-wide and [22].

The most typical way that the periodontal tissues and alveolar bone respond to the trauma is by forming an apical granuloma. Despite the encouraging results associated with pulpotomy, several knowledge gaps persist in the literature. For instance, while studies have predominantly focused on the immediate and short-term outcomes of pulpotomy, there is a lack of comprehensive long-term data assessing the durability of pulp vitality and the incidence of subsequent complications. Future research should aim to establish standardized criteria for case selection and outcome assessment in VPT to facilitate comparisons across studies. Another promising area for future exploration is the comparative effectiveness of pulpotomy procedures in different demographic groups (*e.g.*, age, systemic health status) and the potential influence of patient-specific factors, proper diagnosis, operator experience and the choice of material can significantly influences the outcomes of pulpotomy procedure.

## Conclusion:

Pulpotomy is a viable alternative to traditional root canal treatments. This is particularly true when using advanced materials that enhance healing and vitality preservation. This suggests that the integration of new materials in clinical practice can significantly optimize treatment outcomes.

## References

[1] Zhang M (2022). Sci Rep..

[2] Cvek M (1978). J Endod..

[3] Zhu L (2024). BMC Oral Health..

[4] Lin G.S.S (2024). Children (Basel)..

[5] Duncan H.F (2023). Int Endod J..

[6] Duncan H.F (2019). Int Endod J..

[7] Fuks A.B (2008). J Endod..

[8] Alqaderi H.E (2014). J Dent..

[9] Ricucci D (2014). J Endod..

[10] Ricucci D (2019). J Dent..

[11] Galler K.M (2021). Int J Mol Sci..

[12] Sadaf D (2020). Cureus..

[13] Ramani A (2022). Int Endod J..

[14] Jassal A (2023). Int Endod J..

[15] Taha N.A (2023). J Endod..

[16] Galani M (2017). J Endod..

[17] Alqaderi H (2016). J Dent..

[18] Sadaf D, Ahmad M.Z (2014). Int J Biomed Sci..

[19] Ramsay M.E.A (2000). Proc (BaylUniv Med Cent)..

[20] Peterson M.D (2021). Neurol Clin Pract..

[21] Rechenberg D.K (2016). PLoS One..

[22] Ather A (2022). Sci Rep..

